# Bioinformatics approach to identify the hub gene associated with COVID‐19 and idiopathic pulmonary fibrosis

**DOI:** 10.1049/syb2.12080

**Published:** 2023-10-09

**Authors:** Wenchao Shi, Tinghui Li, Huiwen Li, Juan Ren, Meiyu Lv, Qi Wang, Yaowu He, Yao Yu, Lijie Liu, Shoude Jin, Hong Chen

**Affiliations:** ^1^ Department of Respiration The Fourth Affiliated Hospital of Harbin Medical University Harbin Medical University Harbin Heilongjiang China; ^2^ Department of Respiration Hainan Cancer Hospital Haikou Hainan China; ^3^ Department of Respiration The Second Affiliated Hospital of Harbin Medical University Harbin Medical University Harbin Heilongjiang China

**Keywords:** big data, bioinformatics

## Abstract

The coronavirus disease 2019 (COVID‐19) has developed into a global health crisis. Pulmonary fibrosis, as one of the complications of SARS‐CoV‐2 infection, deserves attention. As COVID‐19 is a new clinical entity that is constantly evolving, and many aspects of disease are remain unknown. The datasets of COVID‐19 and idiopathic pulmonary fibrosis were obtained from the Gene Expression Omnibus. The hub genes were screened out using the Random Forest (RF) algorithm depending on the severity of patients with COVID‐19. A risk prediction model was developed to assess the prognosis of patients infected with SARS‐CoV‐2, which was evaluated by another dataset. Six genes (named NELL2, GPR183, S100A8, ALPL, CD177, and IL1R2) may be associated with the development of PF in patients with severe SARS‐CoV‐2 infection. S100A8 is thought to be an important target gene that is closely associated with COVID‐19 and pulmonary fibrosis. Construction of a neural network model was successfully predicted the prognosis of patients with COVID‐19. With the increasing availability of COVID‐19 datasets, bioinformatic methods can provide possible predictive targets for the diagnosis, treatment, and prognosis of the disease and show intervention directions for the development of clinical drugs and vaccines.

## Graphical abstract

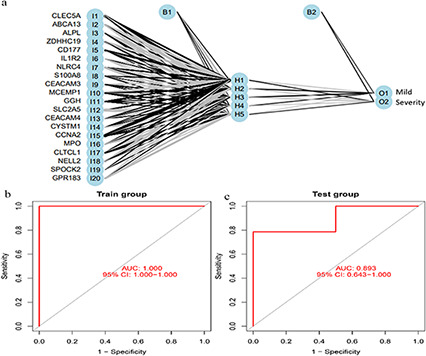

## 1 INTRODUCTION

The coronavirus disease 2019 (COVID‐19) caused by severe acute respiratory syndrome coronavirus 2 (SARS‐CoV‐2) is one of the largest worldwide public health emergencies. The rapid spread of SARS‐CoV‐2, the emergence of variants, and the repeated outbreaks of COVID‐19 in various parts of the world have affected countries across the globe. To date, there are more than 527 million confirmed COVID‐19 cases worldwide, and the total number of deaths due to COVID19 is more than 6.29 million. The ability to study SARS‐CoV‐2 in record time and bring diagnostic, treatment, vaccination, and control options into the public domain are the result of the combined efforts of scientists, governments, pharmaceutical industries, and healthcare systems. Much of this research has been propelled by big data. Bioinformatics has played a key role in the study of COVID‐19 via the storage, search, and analysis of big data to elucidate the biological significance of the genomics and proteomics of SARS‐CoV‐2, thus greatly reducing the experimental research time. Moreover, researchers can quickly find directions, promote progress, and understand the recent and long‐term trends of epidemics via the exchange of information in public databases, and relevant government departments can communicate timely decisions and eliminate public pertaining to about SARS‐CoV‐2 research.

The first gene sequencing of SARS‐CoV‐2 was published in GenBank on 10 January 2020 [1]. The origin of the virus and the relevance of its variants were further analysed using RNA sequencing (RNA‐Seq), which revealed that SARS‐CoV‐2 belongs to the genus β‐coronavirus and that it might have originated from bats [2]. RNA‐Seq also plays an important role in diagnosis, with Polymerase Chain Reaction having been used as the most common means of diagnosing COVID‐19 and the gold standard for confirming SARS‐CoV‐2 infection. Furthermore, RNA‐Seq provides insights into the type of the specific variant and traces its source and transmission route. In terms of treatment, by identifying the hub genes and analysing the proteomic structure of virus, the efficacy of virus‐related targeted drugs can be predicted and novel drugs can be explored in a timely manner. Compared with conventional methods, bioinformatics analysis can greatly reduce the study time, lower the study capital costs, and improve the safety and efficacy of the drug. In the area of immunisation, the development of new vaccines is one of the most effective methods to control the spread and reduce the severity of the disease. Hence, several research institutions around the world are attempting to develop safe and effective vaccine research protocols and conduct clinical trials in the shortest possible time. Bioinformatics plays a pertinent role in vaccine research, including reverse vaccinology and structural vaccinology [3]. RNA‐Seq analysis of samples from individuals with COVID‐19 can help predict the epitopes of immune cells, which can be used for vaccine structural design, protein blotting analysis, and efficacy assessment [4]. In conclusion, bioinformatics can provide an overall understanding of the pathogenesis, diagnosis, treatment, vaccine development, and prediction of disease regression.

Pulmonary fibrosis (PF) is a pathological state of irreversible lung damage due to the excessive deposition of extracellular matrix, including collagen. Interstitial lung disease (ILD), as a group of diffuse lung diseases, mainly involve the pulmonary interstitium and alveolar spaces. Different types of ILD have varying degrees of PF, while idiopathic pulmonary fibrosis (IPF) is considered the most representative type of PF. Approximately 17.2%–31% of patients will progress to acute respiratory distress syndrome (ARDS) after SARS‐COV‐2 viral infection [5]. PF is one of the most common complications of ARDS and is characterised histologically by diffuse alveolar damage. Although PF is one of the major complications of COVID‐19 and deserves more attention. An elderly female patient without underlying lung disease died due to severe fibrosis in both lungs after SARS‐CoV‐2 infection. High resolution computed tomography (HRCT) of the patient showed extensive fibrotic lesions in the lung, and pathologic biopsy of the lungs showed significant fibrotic changes with bronchial remodelling and bronchiectasia [6]. Han et al. [7] showed in a 6‐month follow‐up survey that fibrosis developed in the lungs of patients with COVID‐19 not only during the course of infection, but in more than one‐third of critically ill patients after discharge from the hospital. The exact pathogenesis of fibrosis after SARS‐CoV‐2 infection is not fully understood and further clinical studies are needed. The disease prognosis in patients with COVID‐19 has raised concerns on the mechanisms by which COVID‐19 and IPF act concertedly.

In the field of bioinformatics research, high‐throughput sequencing technology presents obvious advantages and can be used for comprehensive RNA‐Seq analysis of different species to provide new ideas and directions for research by identifying the differentially expressed genes (DEGs) and analysing the possible pathogenesis. A previous studies has demonstrated that severe acute respiratory syndrome‐related coronavirus showed better results in gene expression analysis using high‐throughput RNA‐Seq [8]. In addition to the clinical phenotype, we need to further understand the underlying molecular mechanisms and hub genes. The aim of this study was to determine whether there is a synergistic effect of SARS‐CoV‐2 to IPF patients by transcriptomic analysis of severe SARS‐CoV‐2 infection and IPF patients. Hub genes were identified to discover the molecular mechanisms in patients with COVID‐19 and IPF, and to provide gene targets for the development of targeted therapeutic strategies.

## 2 MATERIALS AND METHODS

### 2.1 Collection of the dataset

**FIGURE 1:**
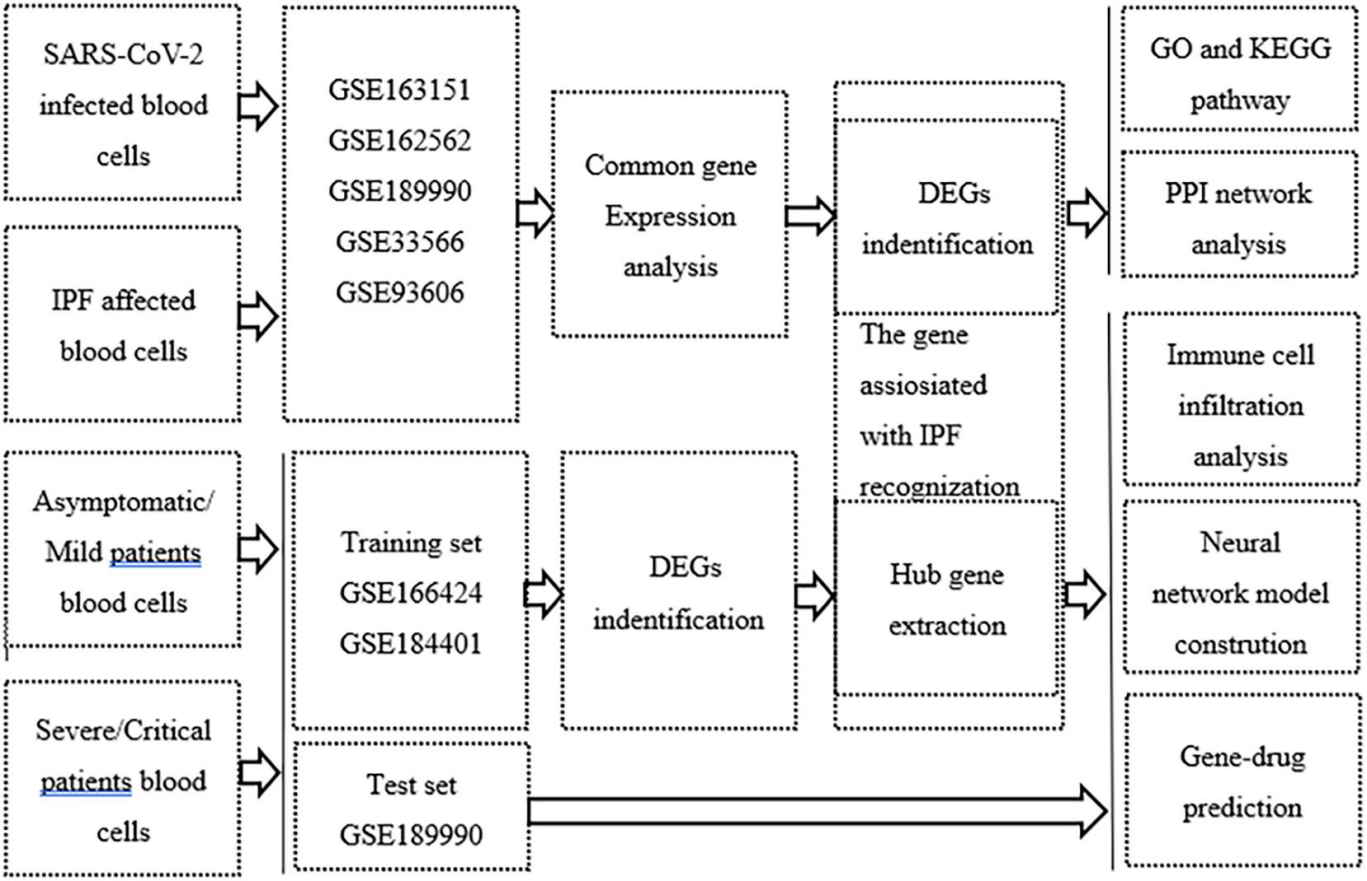
The methodical workflow for the study.

RNA‐Seq analysis of blood samples from patients with COVID‐19 and IPF was performed and the Gene Expression Omnibus (GEO http://www.ncbi.nlm.nih.gov/geo/) database was used to find common mechanisms of action and to investigate the relationship. The study was divided into two parts, and the first part with GEO IDs GSE163151, GSE162562, and GSE189990 were used for the analysis of gene sequencing analysis of patients infected with SARS‐CoV‐2, whereas GSE33566 and GSE93606 were used for the analysis of gene expression in patients with IPF. The experimental group comprised patients with COVID‐19 and IPF, and the control group comprised healthy population. The five datasets were first screened for coexpressed genes. Subsequently, the DEGs were identified, which were used for further studies, including Gene Ontology (GO) and Kyoto Encyclopaedia of Genes and Genomes (KEGG) enrichment analyses. The biological processes of COVID‐19 and IPF coexpression were identified and protein‐protein interaction (PPI) network analysis were performed to extract the coexpressed hub genes. In the second part, GEO accession IDs GSE166424 and GSE184401 were to use to analyse of the training set data and GSE189990 was used to analyse of the test set data. To start with, the hub genes were screened using the Random Forest (RF) algorithm, whereas the intersecting genes were taken with the DEGs identified in the first part to extract the genes associated with PF. Immune correlation analysis was performed for the hub genes, including the scores of immune cells in each subdata set, the analysis of differences between the immune cells in different severity groups, and the correlation analysis of immune cells and the hub genes. Finally, a neural network model was constructed to predict the severity of the disease in the patients based on the hub genes, and the accuracy of the model was predicted using Receiver operating characteristic (ROC) curves. Potentially effective drugs for the treatment of COVID‐19 were screened based on the hub genes. The flow chart for this study is shown in Figure 1.

### 2.2 Data processing

The GEO datasets used in this study were collected from the National Centre for Biotechnology Information (NCBI http://www.ncbi.nlm.nih.gov/geo/; Table 1). The detection of SARS‐CoV‐2 whole‐genome was performed with high‐throughput sequencing methods. GSE163151, GSE162562, and GSE184401 were RNA sequenced on the Illumina NovaSeq 6000 platform [16, 17, 18], whereas GSE189990 and GSE166424 were acquired using RNA‐Seq technology, based on Illumina NextSeq 500 and Illumina NextSeq 4000 platforms, respectively [19, 20]. The IPF sequencing dataset was detected by microarray. GEO database IDs of IPF were GSE33566 and GSE93606, which were provided using Yang IV and Molyneaux PL, respectively [21, 22]. All samples were subjected to gene expression analysis of whole‐‐blood RNA from humans, unifying the study samples to make the study more meaningful. The GSE163151 datasets included 20 healthy samples and 7 samples from individuals affected with COVID‐19. The GSE162562 datasets included 61 healthy samples and 47 samples from individuals affected with COVID‐19. The GSE189990 datasets included four healthy samples and 20 samples from individuals affected with COVID‐19. The IPF dataset with GEO accession ID GES33566 was collected from 30 healthy samples and 93 IPF samples, whereas GSE93606 was obtained from 20 healthy samples and 154 IPF samples. As per the “Diagnosis and Treatment Protocol for Novel Coronavirus Pneumonia (Trial version 7)” published by Chinese General Office of the National Health Commission, the patients were classified as asymptomatic infected, mild, moderate, severe, and critical type based on the severity of the illness [23]. In this study, asymptomatic and mild forms were classified as the mild disease group, whereas the severe and critical forms were categorised as the severe disease group. The training set with GEO accession ID GSE166424 included 32 mild samples and 2 severe samples, and GSE184401 included 21 mild samples and 17 severe samples. The test set (GEO accession ID GSE189990) comprised 2 mild samples and 14 severe samples. All datasets used in this study were downloaded from the GEO public database and were not relevant to any animal or human subjects. Consequently, no additional approval from the ethics board was required.

**TABLE 1:** The summary information for the dataset of this study.

Dataset	Platform	Disease	Sample characteristics	Sample characteristics	Sample characteristics	Sample characteristics	Sample characteristics	Neural networks	Reference
			(Control)	(Total number of patient)	(Asymptomatic/Mild)	(Moderate)	(Severe/Critical)		
GSE163151	GPL24676	Covid19	20	7	0	0	7		[9]
GSE162562	GPL24676	Covid19	61	47	47	0	0		[10]
GSE189990	GPL18573	Covid19	4	20	2	4	14	Test	[11]
GSE166424	GPL20301	Covid19	2	38	32	4	2	Training	[12]
GSE184401	GPL24676	Covid19	0	38	21	0	17	Training	[13]
GSE33566	GPL6480	IPF	30	93	–	–	–		[14]
GSE93606	GPL11532	IPF	20	154	–	–	–		[15]

### 2.3 Identification of the potential molecular mechanisms in COVID‐19 and IPF

#### 2.3.1 Screening out coexpression genes and identifying the DEGs from the COVID‐19 and IPF datasets

The COVID‐19 dataset (GEO accession IDs GSE163151, GSE162562, and GSE189990) was converted to the Transcripts Per Million format, and the Venn diagram was integrated using the limma package combined with the IPF dataset (GEO accession IDs GSE33566, and GSE93606). The intersecting genes were obtained after batch correction using the combat function of the SVA package by considering the batch effects and the biological differences. The DEGs were identified using the limma package on the basis of adj. P. Val/FDR <0.05 and |logFC| ≥ 0.3. The DEGs were then visualised using the pheatmap package. Finally, differential gene expression analysis was performed on the five datasets using the edgeR package and the limma package.

#### 2.3.2 GO and KEGG pathway enrichment analysis

Gene enrichment analyses were used to identify the biological processes and the locations of the chromosomes in different associated diseases and to elucidate their biological significance [24]. The GO database is a structured standard biological model built in 2000, which covers cellular components(CCs), molecular functions(MFs), and biological processes(BPs) [25]. In the KEGG database, the genomic, chemical, and functional information is combined for several organisms, to identify functional and metabolic pathways [26]. The DEGs were identified for the GO and KEGG enrichment pathways via the clusterProfiler package, org.Hs.eg.db package, enrichplot package, and the ggplot2 package in R language, and the coexpressed pathways were extracted for COVID‐19 and IPF.

#### 2.3.3 PPI network analysis

PPI, which is composed of interactions between proteins and genes, was used to analyse processes, such as biological information transfer, energy metabolism and cell cycle regulation, with biological significance [27]. In this study, PPI networks were generated using the String (https://string‐db.org) online analysis software to describe the interactions of DEGs between COVID‐19 and IPF, and the confidence measure was set to 0.400. Subsequently, to visualise the network more clearly and to identify the hub proteins, Cytoscape (3.9.1) software was used to analyse the network and reconstruct the PPI network based on the degree values. Cytoscape is a software that combines security and flexibility to integrate genetic data, such as those of proteins and genes [28].

PPI networks contain nodes and edges that represent PPIs, where the more connected nodes represent the hub proteins. CytoHubba (http://apps.cytoscape.org/apps/cytohubba) is a plugin in Cytoscape for central or potential or target elements of biological networks based on various network characteristics and can extract hub gene, which includes 11 methods for the topological analysis of networks in cytohubba [29]. Maximal Clique Centrality (MCC) algorithm is the most commonly used. In this study, the top 10 hub genes were screened using MCC algorithm, which in turn helped in identifying the important modules coexpressed by COVID‐19 and IPF from the PPI network.

### 2.4 Correlation analysis based on the different severity groups of COVID‐19

#### 2.4.1 Extraction of the hub genes in different severity groups of COVID‐19 using the RF algorithm

RFs are ensemble classifiers that combine multiple decision trees [30], which are themselves a classifier, but are not stable enough for data processing and are prone to overfitting. RFs build a forest of decision trees, and each tree is a subset based on a different feature of the data, thus improving the stability of data processing. Classification of data based on the weight of each decision tree has been successfully applied in several fields for processing and analysing complex data [31]. In this study, the COVID‐19 datasets (GEO accession IDs GSE166424, and GSE184401) were integrated, and the DEGs in the severe and mild disease groups were identified using the limma package. The point with the smallest cross‐validation error rate was found by the randomforest package. The number of trees corresponding to this point was found, and the top 20 genes with the highest importance scores were selected to obtain the hub genes for different severity groups of COVID‐19. Subsequently, the hub genes in the training and test sets that were differentially expressed in the mild and severe disease groups was statistically analysed using the limma package and the ggpubr package, respectively.

#### 2.4.2 Immune cell infiltration analysis

The relationship between the different severity groups of COVID‐19 and immune cells was examined. First, the immune cell scores of the different severity groups were evaluated based on the single‐sample Gene Set Enrichment Analysis (ssGSEA) algorithm using the reshape2, ggpubr, limma, gseabase, and GSVA packages in R language, and immune cell heatmaps were drawn with the pheatmap package. Second, the immune cells were analysed for statistical differences between the mild and severe groups, and violin maps were drawn using the vioplot package. Finally, the correlation of immune cells with the hub genes was analysed and the results were visualised using limma, reshape2, tidyverse, and ggplot2 packages.

#### 2.4.3 Construction of the artificial neural networks based on the hub genes

The artificial neural network is a multilayer feed‐forward neural network that incorporates a back‐propagation algorithm, and its structure includes the input layer, hidden layer, and output layer [32].The advantages of the network are high accuracy, fault tolerance, and fast information processing for complex data. In this study, the scores of the hub genes were calculated using the limma package, and the artificial neural network model was constructed using the neuralnet and NeuralNetTools packages. The hidden layer was obtained based on the scores and weights of the hub genes, and the output layer was obtained according to the weight value of the hidden layer, that is, the group of patients with different severities of COVID‐19. The accuracy of the neural network was predicted by calculating the area under the ROC curve using the pROC packages.

#### 2.4.4 Prediction of drug candidates based on the hub genes

Screening for drugs that may be effective in the treatment of COVID‐19 according to the varying severities of the hub gene was one of the important aspects of this study. Enrichr (https://amp.pharm.mssm.edu/Enrichr/) was a tool for genome‐wide gene enrichment analysis [33]. Candidate drug molecules were predicted using the Drug Signatures database (DSigDB), which was obtained via the Enrichr platform and consisted of 22,527 gene sets. The top 10 drugs were extracted based on the adj. *P*. Val <0.01 to provide a possible theoretical basis for clinical treatment.

### 2.5 Statistical analysis

All statistical analyses were performed using R software (version 4.1.3), and *p* < 0.05 was considered statistically significant.

## 3 RESULTS

### 3.1 Analysis of the common pathways between COVID‐19 and IPF

#### 3.1.1 Extraction of coexpression genes and identification of DEGs in COVID‐19 and IPF

**FIGURE 2:**
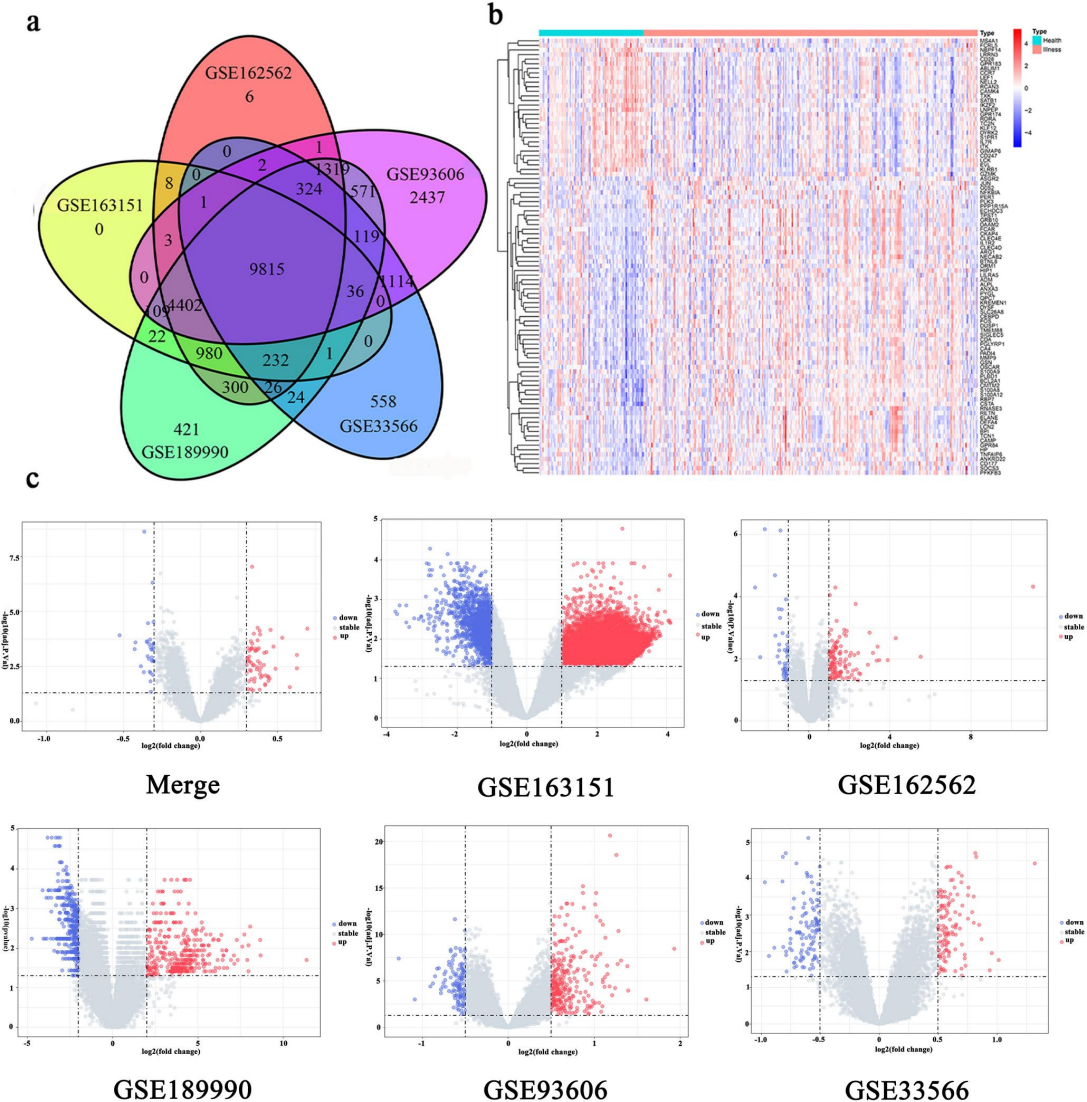
Coexpression genes representation and the DEGs identification of DEGs in patients with COVID‐19 and IPF. (a) Intersecting genes for the 5 datasets of COVID‐19 and IPF. The 9815 genes were found common from SARS‐CoV‐2 infection and IPF patients. (b) Heatmap displaying the DEGs in the diseased and healthy groups. (c) The volcano plot of differentially expressed genes in the 5 datasets after the merging and differentially expressed genes in each data set. Red dots represent upregulated genes, blue dots represent downregulated genes.

Upon extracting the coexpressed genes in the five datasets, the Venn diagram showed that a total of 9815 genes were coexpressed (Figure 2a). The DEGs were identified using the limma package, and 95 DEGs were obtained based on the criteria adj.P.Val/FDR <0.05 and |logFC| ≥ 0.3, of which there were 65 upregulated genes and 30 downregulated genes (Figure 2b). The volcano plot was drawn for the identified DGEs of the three COVID‐19 datasets using the edgeR package based on *P*.Value < 0.05,whereas for the identified DEGs of the two IPF datasets, the limma package was used based on adj.*P*.Val/FDR <0.05 and |logFC| ≥ 0.5 (Figure 2c).

#### 3.1.2 GO and KEGG enrichment analyses of DEGs

**FIGURE 3:**
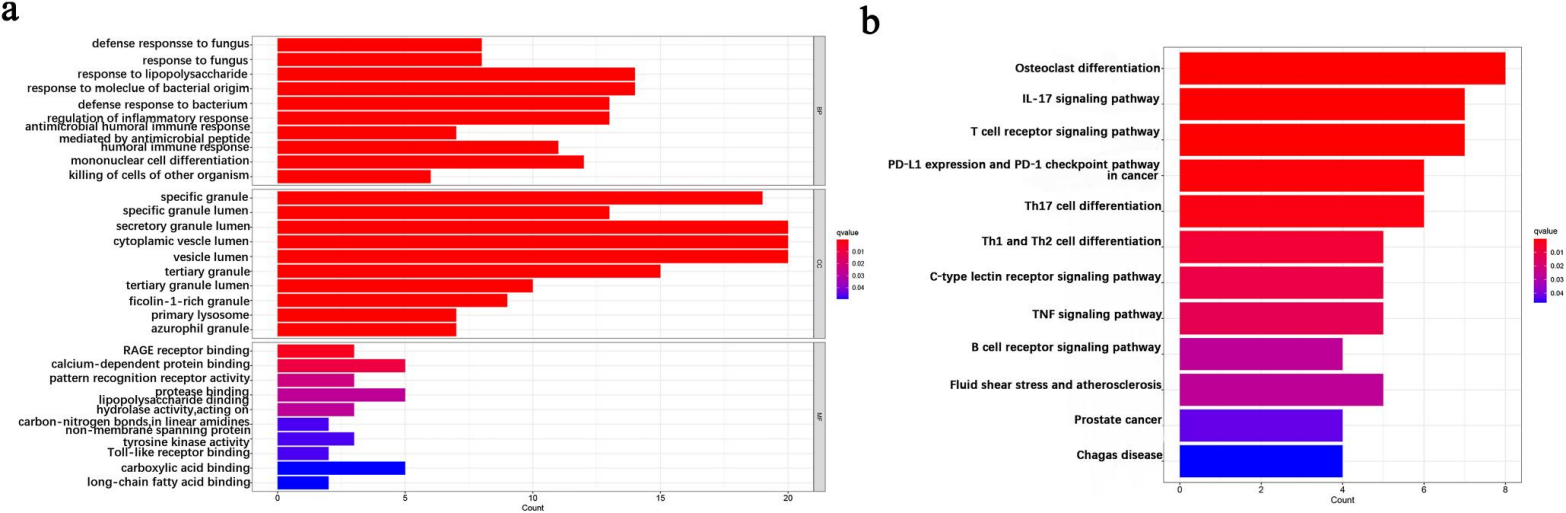
GO and KEGG enrichment analyses for common differentially expressed genes. (a) Scoring results of GO enrichment from BP, CC, and MF to understand differential gene enrichment, the redder the colour, the more significant the enrichment. The higher the number of genes, the higher the enrichment score in a certain ontology. (b) Scoring results of KEGG enrichment analyses. The results of the pathway terminology were determined through a composite score.

To further investigate the functions and pathways of DEGs, GO and KEGG enrichment analyses were performed. There were three top‐level term classes in GO: BPs, CCs and MFs (Table S1, Figure 3a). In BPs, the DEGs in COVID‐19 and IPF were mainly enriched in response to lipopolysaccharide (LPS) and molecular response to bacterial origin. The DEGs were chiefly located in the CCs of secretory granule lumen, cytoplasmic vesicle lumen, and vesicle lumen, and the main MFs of these DEGs were found to bind to calcium‐dependent binding proteins. KEGG analysis was mainly related to osteoclast differentiation, interleukin‐17 (IL‐17) signalling pathway, and T cell receptor signalling pathway (Table S2; Figure 3b). The enrichment results demonstrated that the upregulated and downregulated DEGs can affect various biological functions.

#### 3.1.3 Extraction of hub genes via PPI networks.

**FIGURE 4:**
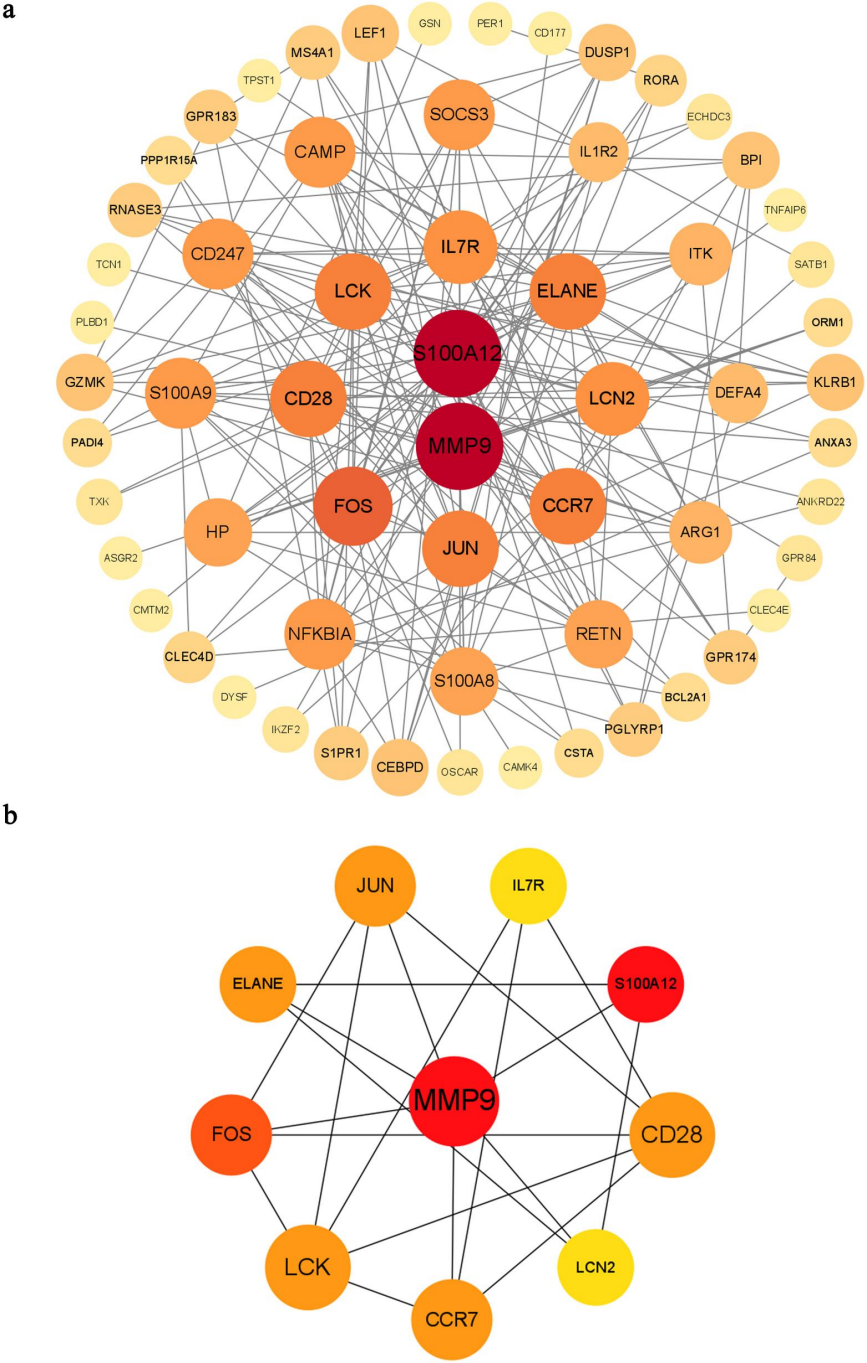
The PPI network identifying the expression of DEGs shared by COVID‐19 and IPF. (a) The PPI network identifying the expression of DEGs shared by COVID‐19 and IPF. In the figure, the circle nodes represent DEGs and edges represent the interactions between nodes. MMP9 and S100A12 are highlighted in red colour as the two hub genes are common among the five datasets. (b) Construction of significant modules from the PPI network using the Cytohubba plugin, redder colours represent higher scores of genes.

PPI networks were constructed to reveal the association of DEGs using the String online analysis software and visualised using the Cytoscape software. In this study, the PPI network consisted of 95 nodes with 186 edges (Figure 4a). The top 10 highest scoring hub genes, namely MMP9, S100A12, FOS, CCR7, CD28, ELANE, LCK, JUN, IL7R, and LCN2, were identified from the PPI network in combination with the Cytohubba plugin of Cytoscape. Significant modules were constructed based on the hub genes (Figure 4b).

### 3.2 The different severity of COVID‐19 associated analysis

#### 3.2.1 Identification of the hub genes in different severity groups of COVID‐19

**FIGURE 5:**
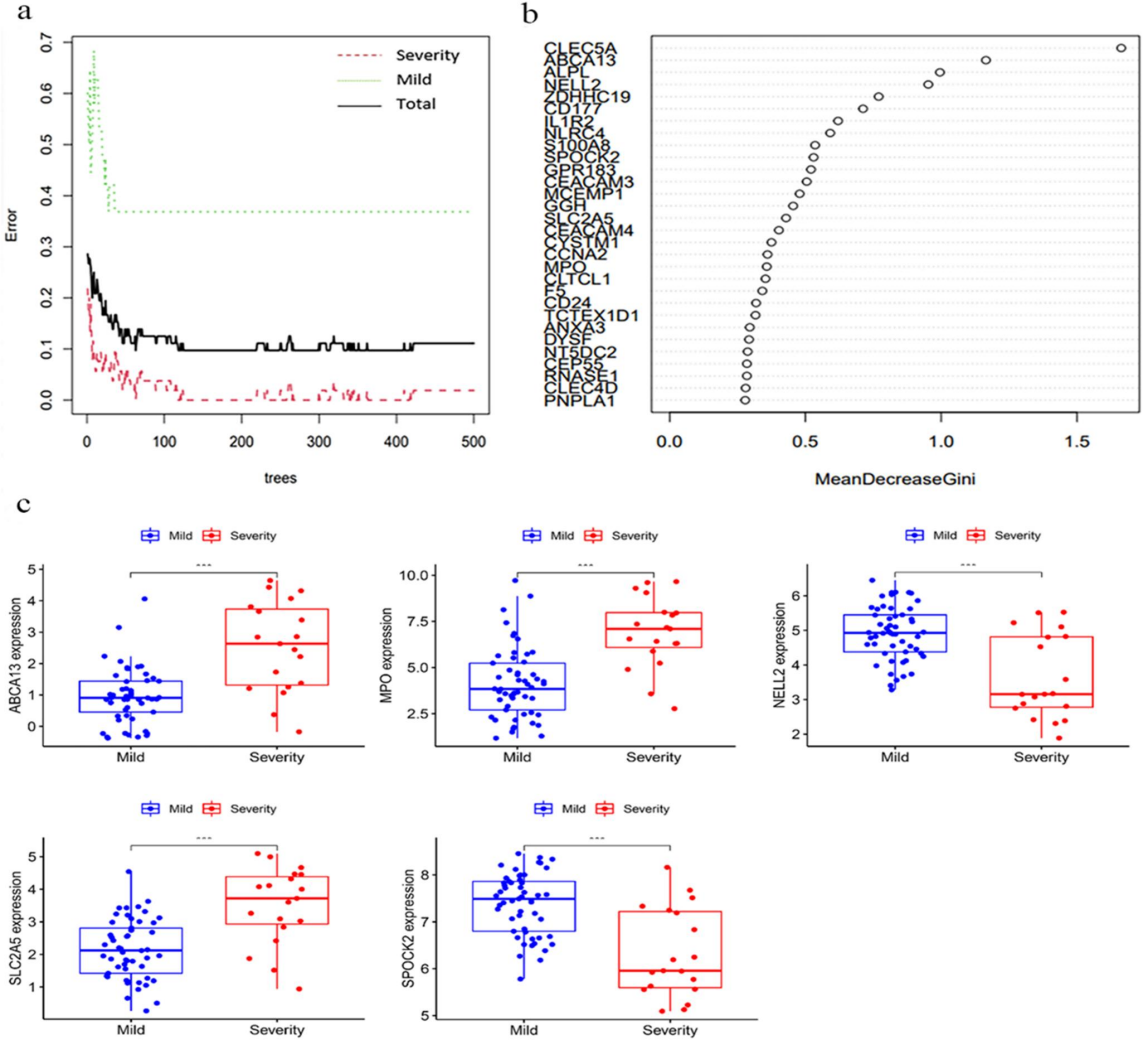
Screening the hub genes according to different severity of patients with COVID‐19. (a) Identification of the disease hub genes by random forest trees algorithm, with the *X*‐axis representing the number of trees and the *Y*‐axis representing the error of cross‐validation. (b) Top 20 disease hub genes with higher scores in different severity groups of COVID‐19. (c) The hub genes (ABCA13,MPO,NELL2,SLC2A5,SPOCK2) with statistical significance in both the training and test set in the severe (red) and mild (blue) disease groups. *p < 0.05, **p < 0.01, ***p < 0.001, ns, not statistically significant.

The DEGs were filtered using the RF algorithm for the severe and mild disease groups of COVID‐19 datasets (GEO accession IDs GSE166424, and GSE184401). The point with the smallest cross‐validation was identified, and the number of trees corresponding to this point was 63. The top 20 genes with high scores were extracted, and the graphs were drawn (Figure 5a,b). Intersection with the 95 DEGs of COVID‐19 and IPF mentioned above was taken to obtain a total of six genes (Figure S1), namely NELL2, GPR183, S100A8, ALPL, CD177, and IL1R2. These genes were considered to be associated with both disease severity of COVID‐19 and PF in the current study, thus providing biomarker targets for future prediction of disease severity and the assessment of progression to PF. Statistical analysis was performed, and box plots were drawn to determine the presence of differences in the expressions of the hub genes between the training and test sets (GEO accession ID GSE189990) in the mild and severe disease groups. A total of five genes were observed to be statistically significant in both datasets, namely ABCA13, MPO, SLC2A5, NELL2, and SPOCK2 (Figure 5c; Figures S2 and S3).

#### 3.2.2 Analysis of immune cell infiltration in the severe and mild groups of COVID‐19

**FIGURE 6:**
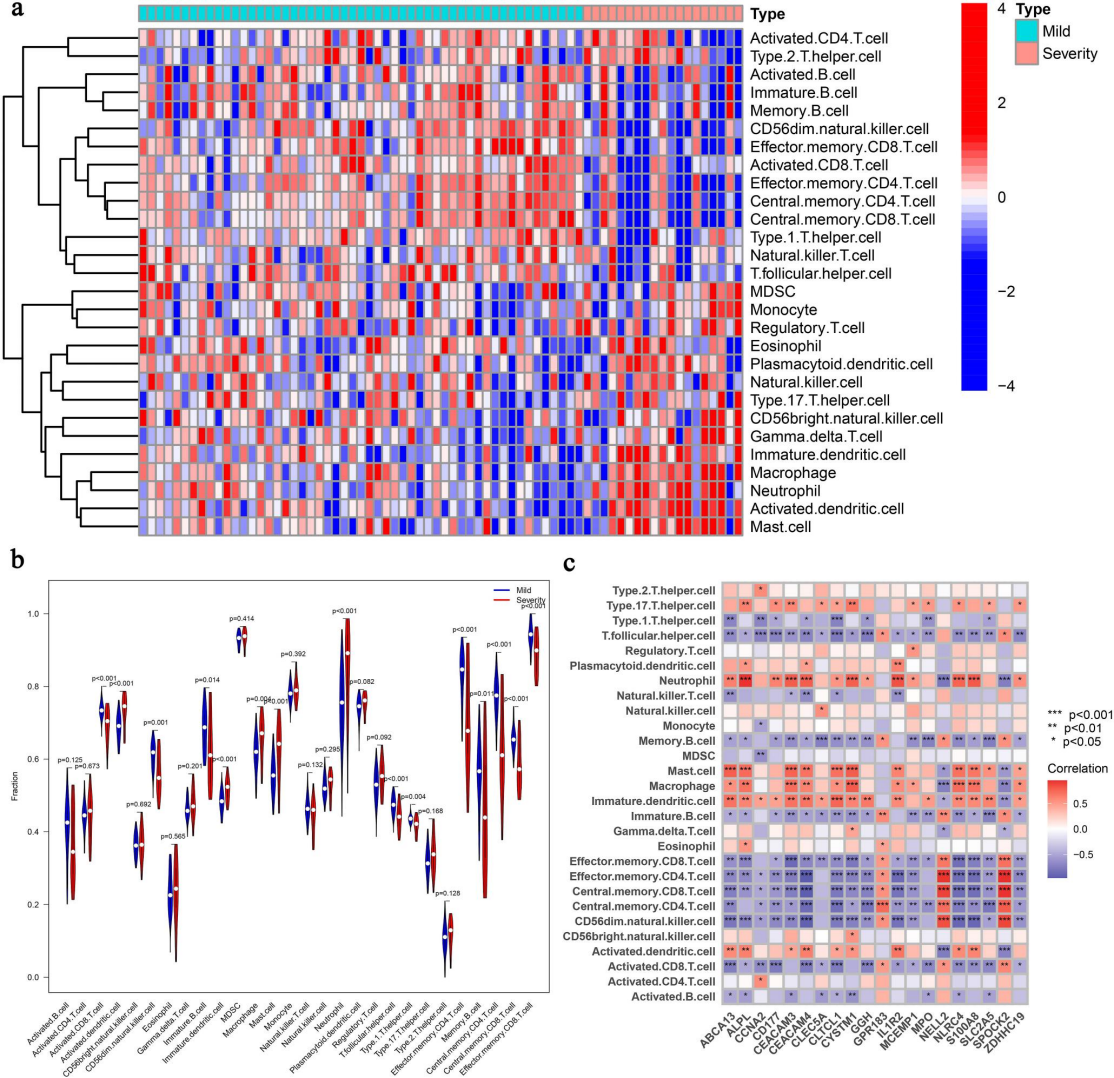
Analysis of immune cell infiltration in line with different severity of patients with COVID‐19. (a) Heat map of immune cell infiltration in the severe and mild disease groups of COVID‐19. (b) The different infiltration of immune cells between the severe and mild disease groups, with horizontal coordinates representing immune cell names and vertical coordinates representing immune cell scores. (c) Relevance of immune cells and disease hub genes with red representing positive correlation and blue representing negative correlation, *p < 0.05, **p < 0.01, ***p < 0.001, ns, not statistically significant.

SARS‐CoV‐2 virus infection activates the host immune system, thereby leading to inflammatory cell infiltration and the release of proinflammatory cytokines. Therefore, it is important to understand the differences in the immune cell infiltration of patients with varying severities of the disease to obtain possible ideas for clinical intervention and treatment. Immune cell infiltration analysis was performed using the ssGSEA algorithm for patients in the severe and mild groups, and a total of 28 immune cells were obtained (Figure 6a). Analysis using heatmap indicated that MDSC, Macrophage, Regulatory.T.cell, CD56bright.natural.killer.cell, Gamma.delta.T.cell, Immature.dendritic.cell, Neutrophil, Activated.dendritic.cell, and Mast.cell were significantly infiltrated in the severe patients group. Subsequently, immune cell infiltration in the severe and mild groups was assessed using violin plot analysis. According to *p* < 0.05, it was found that Activated.CD8.T.cell, Activated.dendritic.cell, CD56dim.natural.killer.cell, Immature.B.cell, Immature.dendritic.cell, Macrophage, Mast.cell, Neutrophil, T.follicular.helper.cell, Type.1.T.helper.cell, Effector.memory.CD4.T.cell, Memory.B.cell, Central.memory.CD4.T.cell, Central.memory.CD8.T.cell, and Effector.memory.CD8.T.cell, a total of 15 cells were statistically significant (Figure 6b). Finally, analysis of the correlation between immune cells and disease hub genes revealed statistical significance based on *p* < 0.05 (Figure 6c).

#### 3.2.3 Construction of a neural network model to predict the severity of the COVID‐19

**FIGURE 7:**
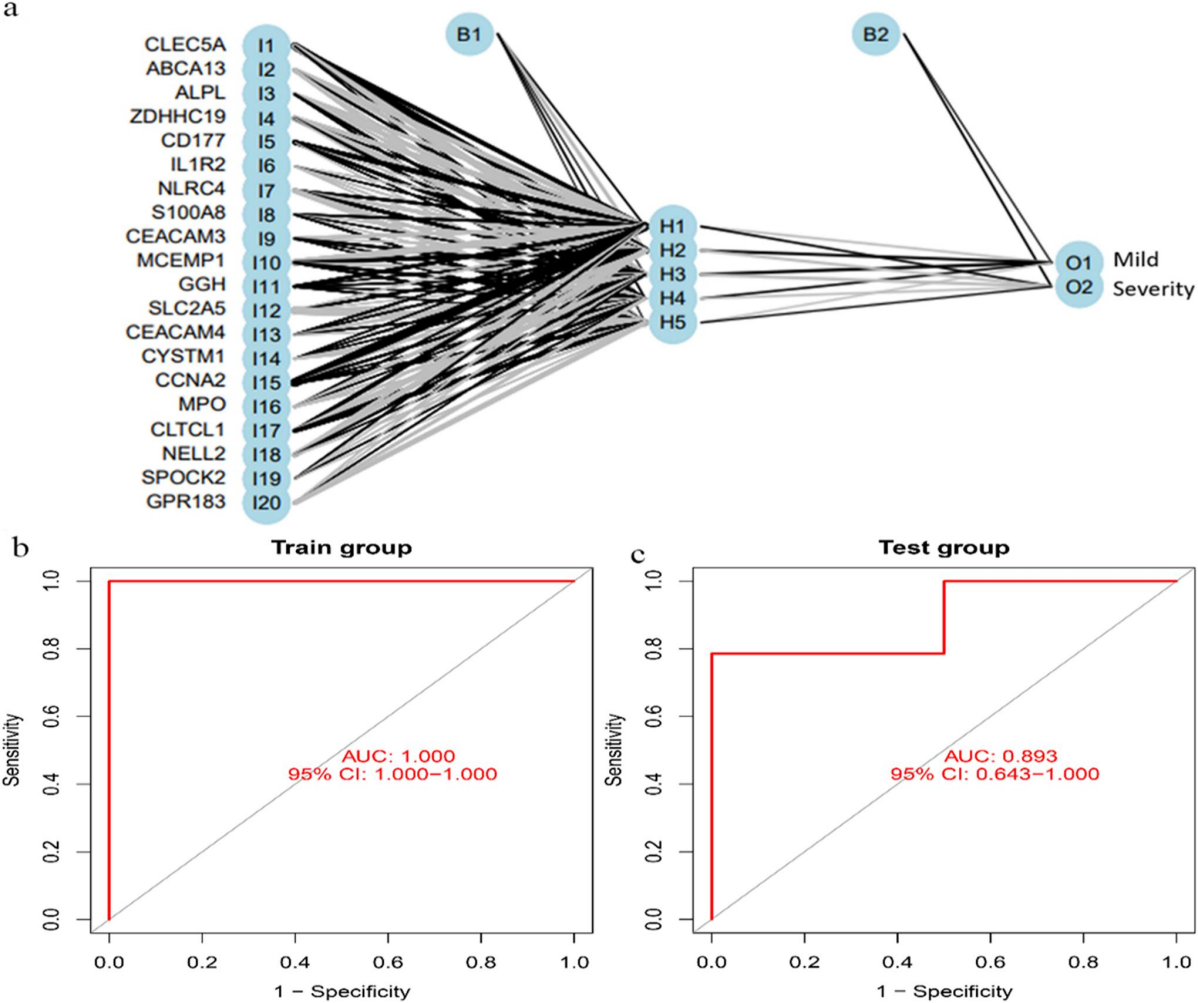
Building neural network models to predict patient prognosis based on the hub genes. (a) Hub gene construction neural network model. (b) ROC analysis for predicting the accuracy of the training set model. (c) ROC analysis for predicting the accuracy of the test set model.

The 20 hub genes were input into the neural network model, the hidden layer was obtained based on the weights of the genes, and the patients with severe and mild diseases were distinguished according to the equal weights of the hidden layer (Figure 7a). The predicted models were validated using ROC curve analysis. The Area Under Curve (AUC) value of the training set was 1.0 (Figure 7b), whereas the AUC value of the test set was 0.893 (Figure 7c), which implies that the accuracy of the neural network model is relatively high. The above training indicates that these genes may serve as potential biomarkers for identifying the severity of COVID‐19.

#### 3.2.4 Identification of drug candidates

**FIGURE 8:**
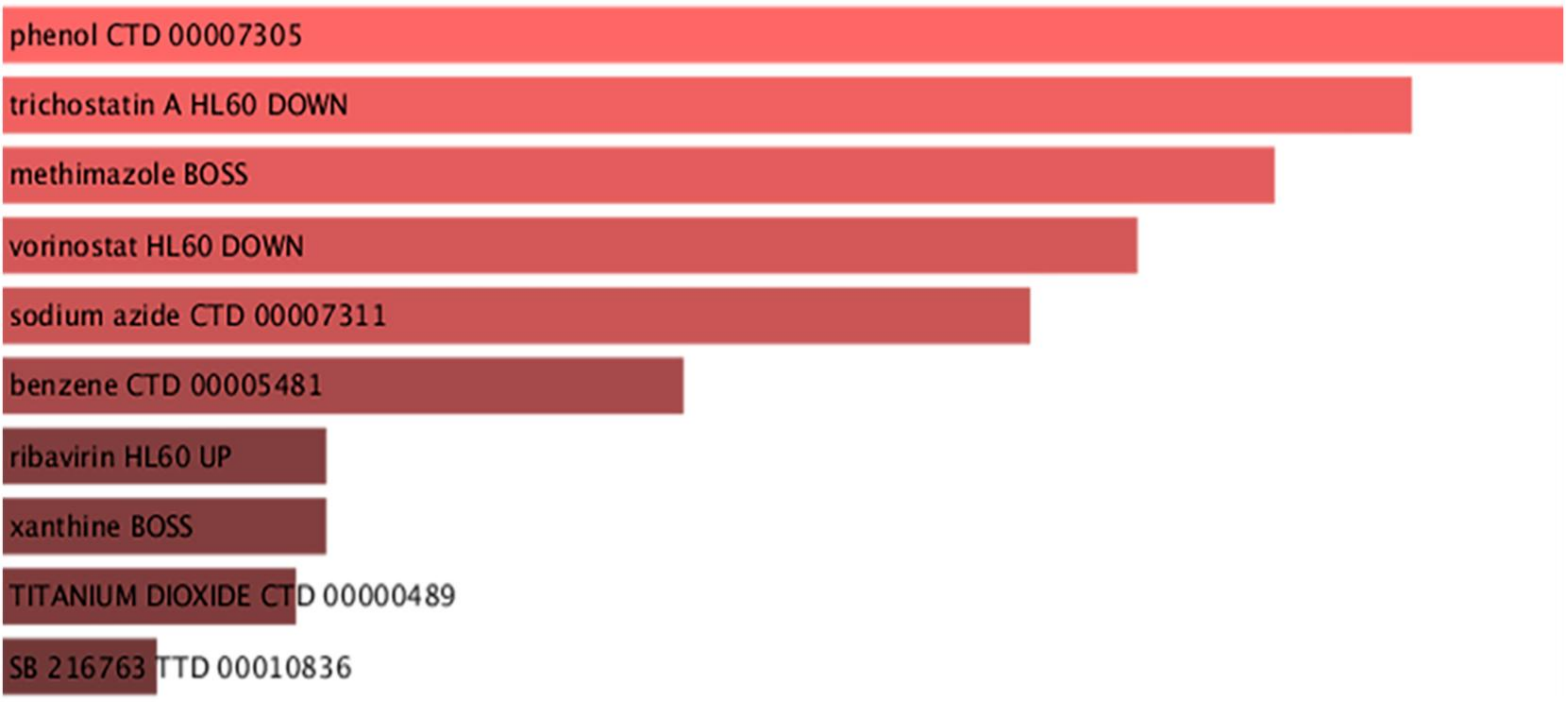
Recommended drugs for SARS‐CoV‐2 infection.

Assessment of gene‐drug interactions is essential to guide clinical treatment. The top 10 drug candidates were extracted from the DSigDB database based on *p* values, namely phenol CTD 00007305, trichostatin A HL60 DOWN, methimazole BOSS, vorinostat HL60 DOWN, sodium azide CTD 00007311, benzene CTD 00005481, ribavirin HL60 UP, xanthine BOSS, TITANIUM DIOXIDE CTD 00000489, and SB 216763 TTD 00010836 (Figure 8). The drugs were found to give good results according to the statistical analysis of the hub genes (Table S3).These drugs have the potential to be effective in the treatment of SARS‐CoV‐2 infection. However, further clinical trials are needed to evaluate the efficacy and study the side effects.

## 4 DISCUSSION

Many studies have found that SARS‐CoV‐2 infection may lead to PF, but the relationship between the occurrence of PF and the duration of viral infection, the level of viral load, and the type of variant, the reversible or irreversible nature of the process, and the specific pathogenesis are not fully understood. Previous studies have found an association between the pathogenesis and pathological process of COVID‐19 and IPF, and that antifibrotic therapy aids in controlling the progression of the disease in patients with severe COVID‐19 [34, 35, 36]. Wendisch et al. found significant similarities in factor expression between macrophages from patients with severe COVID‐19 and those from patients with IPF [37]. Furthermore, SARS‐CoV‐2 infection offers new ideas for the treatment of IPF as it has been shown that the infection affects the prognosis of patients with IPF [38]. In fatal cases of SARS‐CoV‐2 infection, PF is generally identified during autopsy [39]. In addition to lung histological findings at autopsy, radiographic evidence for PF can be seen on chest HRCT scans in both symptomatic and asymptomatic patients after SARS‐CoV‐2 infection [40, 41, 42, 43]. The possible mechanisms leading to PF after SARS‐CoV‐2 infection may be related to the continuous replication of the virus after infection and the activation of the immune response. The activated immune system releases large amounts of proinflammatory factors, chemokines, and profibrotic factors. Moreover, large numbers of inflammatory cells infiltrate the site of infection, further exacerbating the inflammatory response. This study investigated the gene expression associated with COVID‐19, disease severity and IPF based on bioinformatic approach to provide potential target molecular markers for the diagnosis, treatment and vaccine research of SARS‐CoV‐2 infection. Early detection and timely intervention can minimise the number of patients with PF attributed to viral infection.

In this study, the coexpressed genes were extracted from five datasets (GEO accession IDs GSE163151, GSE162562, GSE189990, GSE33566, and GSE93606), and a total of 95 DEGs were identified. GO enrichment analysis revealed that with regard to BPs, the main manifestations were defence response to fungus, response to fungus, response to LPS, response to molecule of bacterial origin, and defence response to bacterium. It was found that the binding of SARS‐CoV‐2 spike(S)‐protein to LPS can lead to an increased inflammatory response by enhancing nuclear factor‐kappa B activation and cytokine response in the monocytes [44]. Among CCs, the DEGs were mainly enriched in specific granule, specific granule lumen, secretory granule lumen, cytoplasmic vesicle lumen, and vesicle lumen. In terms of MFs, the DEGs were predominantly enriched in receptor for advanced glycation end products (RAGE) binding, calcium‐dependent protein binding, pattern recognition receptor (PRR) activity, protease binding, and LPS binding. RAGE, a 35 KDa protein from the immunoglobulin superfamily, is associated with several inflammatory diseases [45]. RAGE expression levels in vivo have been found to be linked to many lung diseases, such as allergic airway inflammation, lung cancer, chronic obstructive pulmonary disease, PF, acute lung injury, cystic fibrosis, and bronchopulmonary dysplasia, as well as pulmonary hypertension associated with the advanced glycation end products and its receptor axis [46, 47]. PRRs are an important part of the host's innate antiviral immune response, and detection of initial viral infection using RNA or DNA depends primarily on the recognition of the viral nucleic acids by host PRRs [48]. Binding to the viruses produces type I and III interferons, which are potent antiviral agents that exert their antiviral effects by inducing interferon stimulated genes, thereby limiting viral entry into the host cell and preventing viral replication [49]. All these enrichment pathways can be used as targets for anti‐SARS‐CoV‐2 studies. In the KEGG functional enrichment analysis, the main targets were osteoclast differentiation, IL‐17 signalling pathway, T cell receptor signalling pathway, programed cell death 1 expression and programed cell death ligand‐1checkpoint pathway in cancer, Th17 cell differentiation, Th1 and Th2 cell differentiation, C‐type lectin receptor signalling pathway and tumour necrosis factor(TNF)signalling pathway. SARS‐CoV‐2 infection may damage the bone, and it was found that patients with severe COVID‐19 had reduced blood calcium and phosphorus levels compared with those who had moderate COVID‐19 [50]. Gao et al. observed that SARS‐CoV‐2 infection affects the bone marrow‐derived macrophages (BMM) in a neuropilin‐1‐dependent manner and inhibits the differentiation of BMM to osteoblasts [51]. This finding is important for cellular and histological view in the early stages of viral infection. Lin et al. uncovered that the SARS‐CoV‐2‐encoded protein open reading frame 8 (ORF8) can act as a major factor that leads to cytokine storm during COVID‐19, and that ORF8 promotes the expression of inflammatory factors primarily through activation of the IL‐7 signalling pathway [52]. De Biasi et al. noted that in patients with COVID‐19, different degrees of immunosuppression existed in the cells at different stages and that the activated cells may be more inclined to differentiate towards the Th17 phenotype when large amounts of inflammatory cytokines are produced in vivo [53]. TNF‐α was found to be an important pro‐inflammatory factor that caused extensive lung tissue damage in a mouse model of SARS‐CoV‐2 infection. Treating with neutralising antibodies against TNF‐α protected mice from mortality during SARS‐CoV‐2 infection, sepsis, and cytokine shock [54]. Selvaraj et al. similarly in constructing a functional enrichment of an early biological model of SARS‐CoV‐2 infection found correlation with the TNF signalling pathway [55].

To gain insights into the biology of the proteomics between COVID‐19 and IPF, a PPI network was established based on DEGs. The hub genes were identified according to degree value, that is, MMP9, S100A12, FOS, CCR7, CD28, ELANE, LCK, JUN, IL7R, and LCN2. Among them, MMP9 and S100A12 were inferred to be the core genes in this network. Paula et al. discovered a significant increase in the expression of MMP‐9 using immunohistochemical methods in a group of patients with COVID‐19 [56]. Furthermore, during the degradation of the extracellular matrix, MMP‐9 activated transforming growth factor‐beta (TGF‐β) by binding to CD44v6, which in turn exacerbated the tissue remodelling process [57]. Lei found that S100A12 was a prominent biomarker for severe influenza and that its expression tended to increase with increasing viral load. In COVID‐19, S100A12 expression was significantly increased in the severe and critical groups of patients and was associated with patient prognosis [58].

COVID‐19 datasets (GEO accession IDs GSE166424, and GSE184401) were used as training sets to identify DEGs by grouping them according to disease severity and filtering out the top 20 scoring hub genes using RF algorithm. Huang et al. observed that patients with severe COVID‐19 were more likely to progress to PF during a 1‐year follow‐up [59]. A neural network model for predicting disease progression and risk assessment in patients was constructed, and the training and test sets (GEO accession ID GSE189990) were evaluated with a relatively high accuracy based on the AUC curve. The 20 hub genes and the 95 DEGs mentioned above were taken to intersect the genes to obtain a total of 6 signature genes, namely NELL2, GPR183, S100A8, ALPL, CD177, and IL1R2. A search of the GeneCards database revealed that S100A8 is associated with both IPF and COVID‐19, while other hub genes still need to be further validated by in vivo and in vitro experiments. S100A8 is a calcium‐ and zinc‐binding protein which plays a prominent role in the regulation of inflammatory processes and immune response. It can induce neutrophil chemotaxis and adhesion. Guo et al. found that S100A8 led to massive activation of neutrophils via toll‐like receptors 4 signalling after SARS‐CoV‐2 infection, and Paquinimod, a specific inhibitor of S100A8, significantly reduced viral load in SARS‐CoV‐2‐infected mice, thereby attenuating their immune response [60]. New drug for ALPL targeting named zinc sulphate is currently in clinical trials for COVID‐19 [61]. In a small study of bronchoalveolar lavage fluid (BALF) from patients with COVID‐19, a significant infiltration of lymphocyte dominated inflammatory cells was seen in the BALF, while CD177 expression was significantly increased in the BALF of critically ill patients with COVID‐19 [62]. These genes can be used as potential biological markers to assess disease severity and progression to PF.

In this study, we found a significant relationship between several immune cell subsets and the severity of COVID‐19, with MDSC, Macrophage, Regulatory.T.cells, CD56bright.natural.killer.cells, Gamma.delta.T.cells, Immature.dendritic.cells, Neutrophil, Activated.dendritic.cells, and Mast.cells being significantly clustered in the group of patients with severe disease. Natural killer (NK) cells are innate effector lymphocytes and are generally divided into cytokine‐producing CD56bright.natural.killer.cell and cytotoxic CD56dim.natural.killer.cell [63]. NK cells not only target and kill the virus‐infected cells but also assist in adaptive T‐cell responses. In patients with NK cell deficiency fulminant viral infections may occur. The present study noted a significant infiltration of CD56bright.natural.killer.cells in the severe disease group, which is consistent with the findings of Maucourant et al. [64]. There is a significant release of inflammatory factors in patients with severe COVID‐19, and the release of these factors may originate from the invasion of alveolar macrophages by SARS‐CoV‐2 via angiotensin‐converting enzyme 2, thus resulting in the release of interleukin‐6 and TGF‐β from alveolar macrophages [65, 66]. In an autopsy report, alveolar macrophages were significantly infiltrated and activated in patients with severe COVID‐19 [67]. In this study too, the ssGSEA method showed that there was an aggregation of alveolar macrophages in the severe disease group. Mast cells can play a critical role in the SARS‐CoV2‐mediated inflammatory response by recognising viral products and synthesising many chemokines and cytokines. Afrin et al. stated that SARS‐CoV‐2 infection induced the excessive activation of mast cells to produce a cytokine storm, which may lead to lung injury and pleural fluid accumulation [68]. A large number of patients with COVID‐19 die from cardiovascular complications, such as pulmonary embolism, sepsis, and multiorgan failure. Studies have signified that mast cells also play an important role in promoting thrombotic disease and that inhibiting their activation can help improve the prognosis of patients with severe sepsis [69]. Neutrophils are innate immune cells that inactivate viruses and produce cytokines to inhibit viral replication, mainly via phagocytosis, degranulation, and the production of neutrophil extracellular traps (NETs) [70, 71]. Veras et al. investigated 32 patients with severe COVID‐19 and documented significantly higher levels of NETs in the plasma and bronchoalveolar lavage fluid [72]. NETs are a double‐edged sword, protecting the body from infection while also triggering a series of inflammatory responses that damage the surrounding tissues, promote microthrombosis, and lead to permanent organ damage in the lungs, cardiovascular system, and kidneys [73]. Hence, targeted drugs using NETs may help alleviate the lung damage caused by excessive inflammatory responses. However, tests are performed in vivo and/or in vitro.

The hub genes extracted based on the severity of COVID‐19 were used to identify drugs that are likely to be effective in the treatment of SASR‐COV‐2. In this study, the top 10 drug candidates were screened from the DSigDB database based on *p* value. Phenolic compounds are secondary metabolites produced by plants and have strong antioxidant activity due to the presence of phenolic hydroxyls. Studies have shown that these compounds also exert anti‐inflammatory, antiviral, antifungal, and anticancer effects [74, 75, 76, 77, 78]. Among phenolic compounds with antiviral activity, resveratrol has been used to offer protection against infection by viruses such as Middle East respiratory syndrome and SARS‐CoV‐2 [79, 80]. Trichostatin A, one of the most common histone deacetylase inhibitors, is an antifungal antibiotic derived from *Streptomyces hygroscopicus* and induces apoptosis in various cancer cells, including leukemia cells [81]. Phenylmethimazole is a derivative of methimazole that can block the production of inflammatory factors and chemokines in the pathogenesis of inflammation to exert anti‐inflammatory effects, and has been shown to be a candidate for treating COVID‐19‐induced inflammatory responses [82]. Vorinostat, as an anticancer histone deacetylase inhibitor, Sinha et al. established that it can upregulate the expression of angiotensin converting enzyme 2, which is the main target of SARS‐CoV‐2 in the host cells, thus providing a new clue for clinical studies [83]. Sodium azide exerts its antiviral effect mainly by inhibiting cytochrome oxidase and blocking the synthesis of influenza A virus in the chick embryo chorioallantoic membrane [84]. Ribavirin inhibits viral replication primarily by disrupting the activity of RNA dependent RNA polymerases (RdRp), a key enzyme in the viral replication cycle. A study showed that ribavirin binds to the RdRp of SARS‐CoV‐2 and interferes with protein synthesis, thereby inactivating it [85]. Acyclic nucleoside phosphonate bearing xanthine as a nucleobase possesses potent antiviral activity and can inactivate herpes viruses and cytomegaloviruses, thus suggesting that xanthine nucleotide analogues could be a promising direction in antiviral drug research [86]. In conclusion, this study hypothesised drugs that may be effective against SARS‐CoV‐2 infection based on the hub gene for COVID‐19 disease severity. However, the information is insufficient for clinical practice, and more in vitro and in vivo experiments are needed to verify the safety and efficacy of these drugs.

The study has several limitations. Firstly, SARS‐CoV‐2 is a novel coronavirus discovered in the last few years, which has been less studied to date, and the small sample size may have biased the results of the study. Second, all samples were subjected to gene expression analysis of whole‐‐blood RNA from humans, whereas lung tissue may be more representative of the pathogenesis of the disease. Third, the present molecular mechanisms and predicted drugs of this article were obtained from public datasets and need to be validated by further in vivo and/or in vitro experiments. Despite the above research limitations, the detection of common pathways and molecular biomarkers in IPF and COVID19 by transcriptome analysis in this study may still provide a theoretical basis for disease development and treatment.

## 5 CONCLUSIONS

Our study analysed the relationship between COVID‐19 and IPF whole genome sequence in the context of RNA‐Seq. First, the results for GO and KEGG enrichment analysis may enhance the understanding of the pathogenesis of COVID‐19. Second, six genes (named NELL2, GPR183, S100A8, ALPL, CD177, and IL1R2) may be associated with the development of PF in patients with severe SARS‐CoV‐2 infection, and S100A8 is one of the most important target genes. Early warning information for the prognosis of patients with COVID‐19 was provided at the gene level. Finally, early detection, early diagnosis, and early treatment of pulmonary fibrosis can help patients improve their quality of life. This study provides a new means of predicting the prognosis and treatment of patients with COVID‐19 and may provide a new direction for immunotherapy.

## AUTHOR CONTRIBUTIONS

**Wenchao Shi**: Conceptualisation; data curation; formal analysis; investigation; methodology; project administration; resources; software; supervision; validation; writing – original draft; writing – review & editing. **Tinghui Li**: Data curation; formal analysis; visualisation. **Huiwen Li**: Formal analysis. **Juan Ren**: Methodology. **Meiyu Lv**: Project administration. **Qi Wang**: Resources. **Yaowu He**: Software. **Yao Yu**: Supervision. **Lijie Liu**: Validation. **Shoude Jin**: Visualisation. **Hong Chen**: Funding acquisition.

## CONFLICT OF INTEREST STATEMENT

The authors declare no conflicts of interest.

## Supporting information

The datasets used to support the findings of this study are included within the article and presented in the references. The raw whole genome expression microarray dataset of COVID‐19 can be obtained from the GEO datasets.Supporting Information S1

## Data Availability

The datasets used to support the findings of this study are included within the article and presented in the references. The raw whole genome expression microarray dataset of COVID‐19 can be obtained from the GEO datasets.
