# Supplementary material for: Bioinformatics approach to identify the hub gene associated with COVID‐19 and idiopathic pulmonary fibrosis
Source: IET Syst Biol. 2023 Oct 9;17(6):336–51. doi: 10.1049/syb2.12080 (PMC10725713; doi:10.1049/syb2.12080)
Supplement: Supplementary file 1 — The datasets used to support the findings of this study are included within the article and presented in the references. The raw whole genome expression microarray dataset of COVID‐19 can be obtained from the GEO datasets.Supporting Information S1 [file SYB2-17-336-s001.docx]

**Supplementary material**

Extraction of the intersecting genes associated with pulmonary fibrosis based on the severity of COVID-19


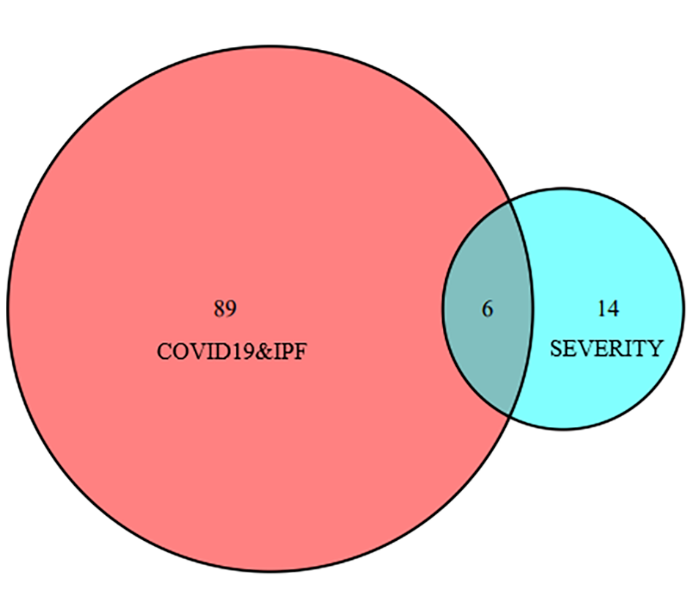


Figure S1: The Venn diagram showing the intersecting genes according to the hub genes screened for different severity of COVID-19 and the DEGs of COVID-19, IPF.

Statistical analysis of the hub gene in the training set


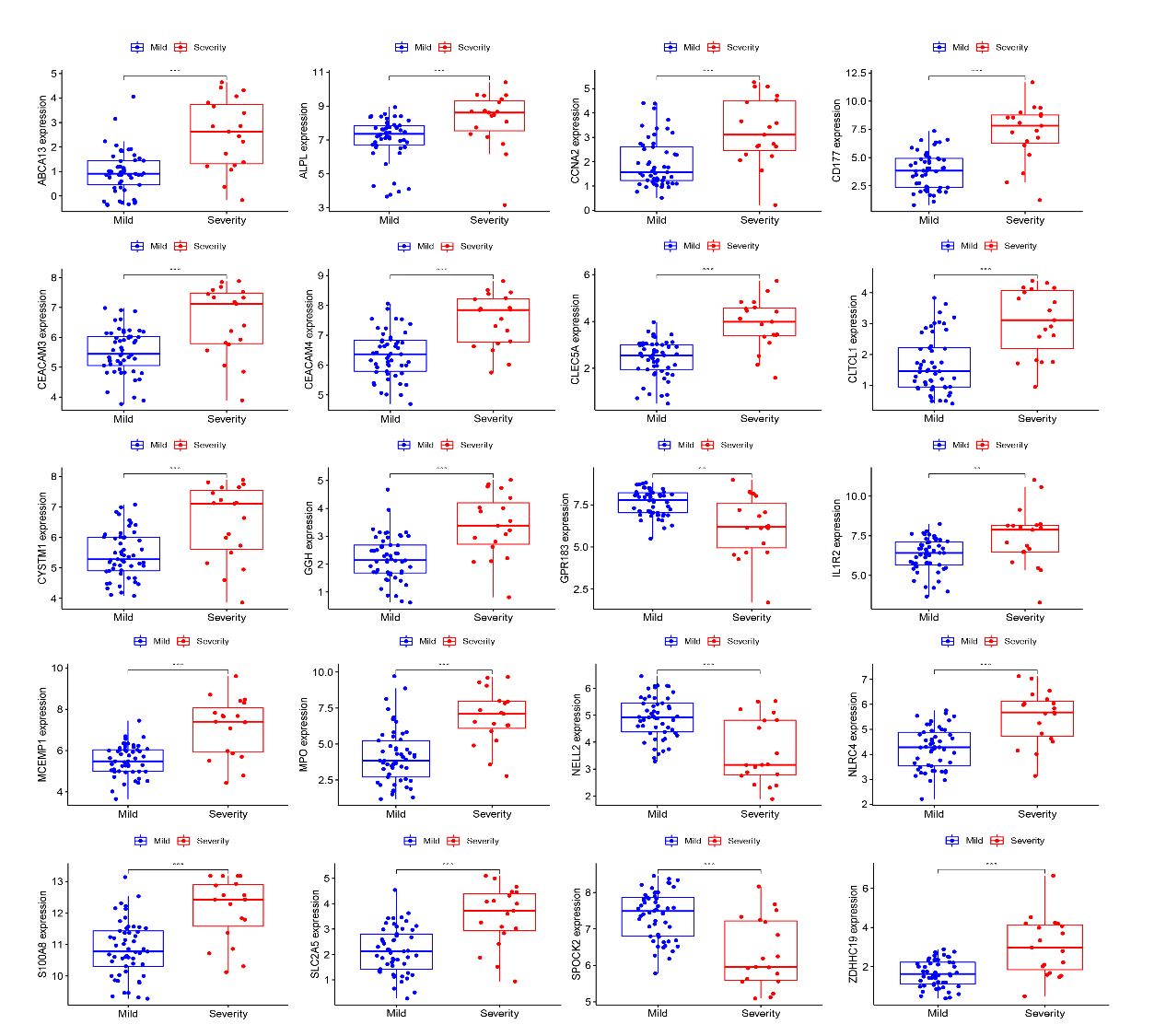


Figure S2: Statistical analysis of the training set in the severe (red) and mild (blue) disease groups for the hub genes. *p < 0.05, **p < 0.01, ***p < 0.001, ns, not statistically significant.

Statistical analysis of the hub gene in the test set


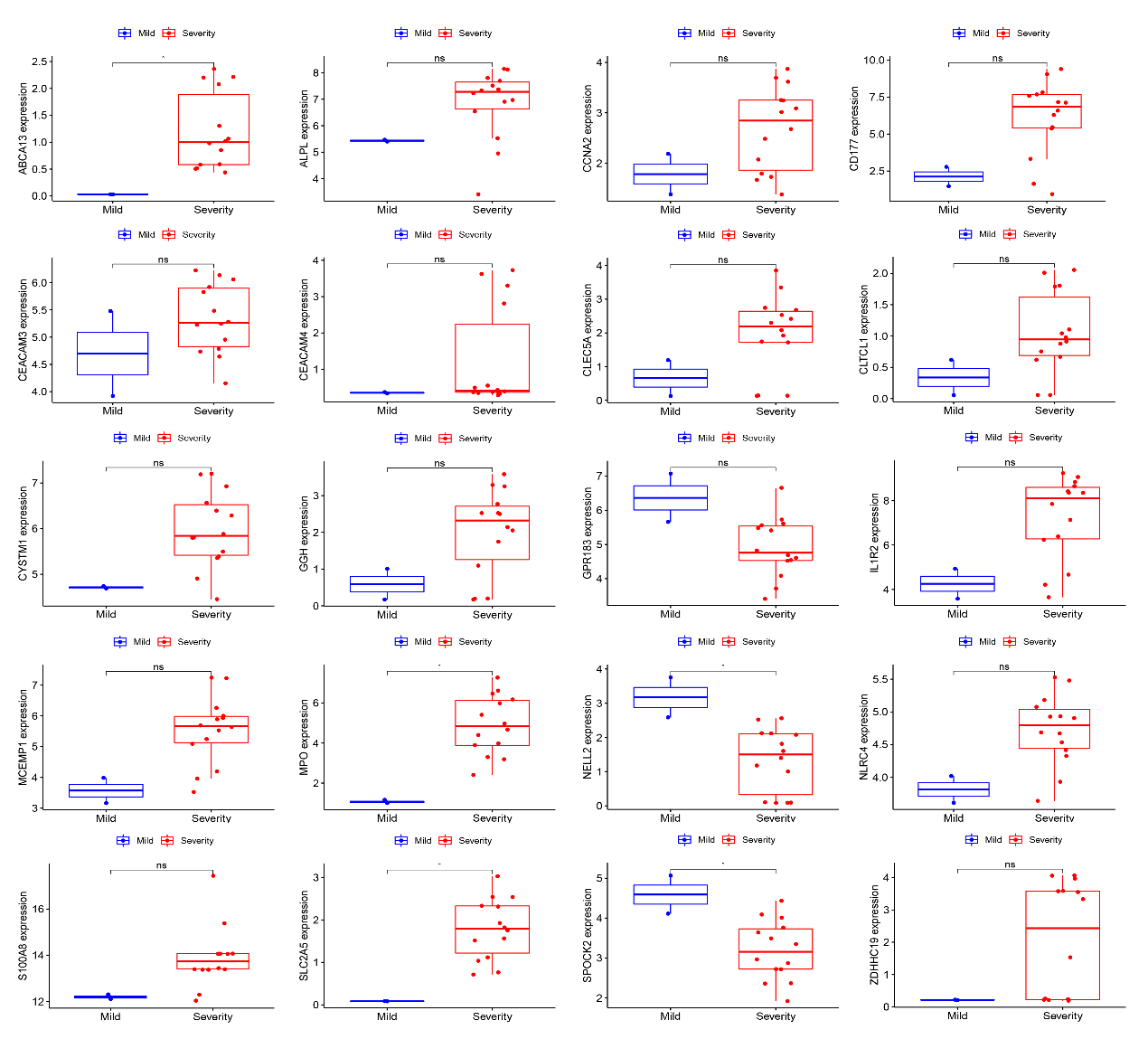


Figure S3: Statistical analysis of the test set in the severe (red) and mild (blue) disease groups for the hub genes. *p < 0.05, **p < 0.01, ***p < 0.001, ns,not statistically significant.

Table S1. Gene ontological analysis of DEGs among COVID-19 and IPF

| ONTOLOGY | ID | Description | Pvalue | GeneCount |
| --- | --- | --- | --- | --- |
| BP | \| GO:0050832 \| \| --- \| | defense response to fungus | 7.27E-11 | 8 |
| BP | \| GO:0009620 \| \| --- \| | response to fungus | 3.95E-10 | 8 |
| BP | \| GO:0032496 \| \| --- \| | response to lipopolysaccharide | 8.14E-10 | 14 |
| BP | \| GO:0002237 \| \| --- \| | response to molecule of bacterial origin | 1.69E-09 | 14 |
| BP | \| GO:0042742 \| \| --- \| | defense response to bacterium | 1.07E-08 | 13 |
| BP | \| GO:0050727 \| \| --- \| | regulation of inflammatory response | 3.39E-08 | 13 |
| BP | GO:0061844 | antimicrobial humoral immune response  mediated by antimicrobial peptide | 9.48E-08 | 7 |
| BP | GO:0006959 | humoral immune response | 3.11E-07 | 11 |
| BP | GO:1903131 | mononuclear cell differentiation | 8.05E-07 | 12 |
| BP | GO:1903131 | killing of cells of other organism | 8.40E-07 | 6 |
| CC | \| GO:0042581 \| \| --- \| | specific granule | 1.58E-21 | 19 |
| CC | \| GO:0035580 \| \| --- \| | specific granule lumen | 2.13E-18 | 13 |
| CC | \| GO:0034774 \| \| --- \| | secretory granule lumen | 5.38E-17 | 20 |
| CC | \| GO:0060205 \| \| --- \| | cytoplasmic vesicle lumen | 6.44E-17 | 20 |
| CC | \| GO:0031983 \| \| --- \| | vesicle lumen | 7.25E-17 | 20 |
| CC | \| GO:0070820 \| \| --- \| | tertiary granule | 1.96E-15 | 15 |
| CC | \| GO:1904724 \| \| --- \| | tertiary granule lumen | 9.89E-14 | 10 |
| CC | \| GO:0101002 \| \| --- \| | ficolin-1-rich granule | 2.67E-07 | 9 |
| CC | GO:0005766 | primary lysosome | 9.88E-06 | 7 |
| CC | GO:0042582 | azurophil granule | 9.88E-06 | 7 |
| MF | \| GO:0050786 \| \| --- \| | RAGE receptor binding | 1.42E-05 | 3 |
| MF | \| GO:0048306 \| \| --- \| | calcium-dependent protein binding | 7.97E-05 | 5 |
| MF | \| GO:0038187 \| \| --- \| | pattern recognition receptor activity | 0.000291 | 3 |
| MF | \| GO:0002020 \| \| --- \| | protease binding | 0.000587 | 5 |
| MF | \| GO:0001530 \| \| --- \| | lipopolysaccharide binding | 0.000595 | 3 |
| MF | GO:0016813 | hydrolase activity, acting on carbon-nitrogen bonds, in linear amidines | 0.001325 | 2 |
| MF | GO:0004715 | non-membrane spanning protein tyrosine kinase activity | 0.001482 | 3 |
| MF | GO:0035325 | Toll-like receptor binding | 0.001585 | 2 |
| MF | GO:0031406 | carboxylic acid binding | 0.001916 | 5 |
| MF | GO:0036041 | long-chain fatty acid binding | 0.001916 | 2 |

Table S2. Pathway enrichment analysis of DEGs among COVID-19 and IPF

| ID | Description | Pvalue | GeneCount |
| --- | --- | --- | --- |
| \| hsa04380 \| \| --- \| | Osteoclast differentiation | 1.46E-06 | 8 |
| \| hsa04657 \| \| --- \| | IL-17 signaling pathway | 2.18E-06 | 7 |
| \| hsa04660 \| \| --- \| | T cell receptor signaling pathway | 4.30E-06 | 7 |
| \| hsa05235 \| \| --- \| | PD-L1 expression and PD-1 checkpoint pathway  in cancer | 2.18E-05 | 6 |
| \| hsa04659 \| \| --- \| | Th17 cell differentiation | 6.54E-05 | 6 |
| \| hsa04658 \| \| --- \| | Th1 and Th2 cell differentiation | 0.000308 | 5 |
| \| hsa04625 \| \| --- \| | C-type lectin receptor signaling pathway | 0.000544 | 5 |
| \| hsa04668 \| \| --- \| | TNF signaling pathway | 0.000763 | 5 |
| \| hsa04662 \| \| --- \| | B cell receptor signaling pathway | 0.001921 | 4 |
| \| hsa05418 \| \| --- \| | Fluid shear stress and atherosclerosis | 0.002003 | 5 |
| \| hsa05215 \| \| --- \| | Prostate cancer | 0.003542 | 4 |
| \| hsa05142 \| \| --- \| | Chagas disease | 0.004242 | 4 |

Table S3. List of the recommended drugs for COVID-19

| Term | Pvalue | Combined Score | Genes |
| --- | --- | --- | --- |
| phenol CTD 00007305 | 1.42E-04 | 310.8477 | CCNA2;ALPL;MPO |
| trichostatin A HL60 DOWN | 1.60E-04 | 105.2771 | CCNA2;IL1R2;GPR183;CLEC5A;SLC2A5 |
| methimazole BOSS | 1.79E-04 | 1063.478 | GGH;MPO |
| vorinostat HL60 DOWN | 1.99E-04 | 97.78538 | CCNA2;IL1R2;GPR183;CLEC5A;SLC2A5 |
| sodium azide CTD 00007311 | 2.17E-04 | 935.4998 | CCNA2;MPO |
| benzene CTD 00005481 | 2.86E-04 | 86.37761 | IL1R2;SPOCK2;CLEC5A;MCEMP1;MPO |
| ribavirin HL60 UP | 3.80E-04 | 646.7588 | IL1R2;CLTCL1 |
| xanthine BOSS | 3.80E-04 | 646.7588 | GGH;MPO |
| TITANIUM DIOXIDE CTD 00000489 | 3.89E-04 | 193.5984 | ALPL;NLRC4;MPO |
| SB 216763 TTD 00010836 | 4.34E-04 | 591.8142 | CCNA2;GPR183 |
